# Pediatric Synovitis, Acne, Pustulosis, Hyperostosis, Osteitis (SAPHO) Syndrome: Diagnostic Challenges and Treatment Approach

**DOI:** 10.7759/cureus.7595

**Published:** 2020-04-09

**Authors:** Niki Kyriazi, Yvonne-Mary Papamerkouriou, Despoina Maritsi, Maria Angela Dargara, John Michelarakis

**Affiliations:** 1 Orthopaedics, Panagiotis & Aglaia Kyriakou Children's Hospital, Athens, GRC; 2 Paediatrics, Panagiotis & Aglaia Kyriakou Children's Hospital, Athens, GRC; 3 Orthopaedics, General Hospital Asklipieio Voulas, Athens, GRC

**Keywords:** sapho syndrome, acne, crmo, biological therapy

## Abstract

Synovitis, acne, pustulosis, hyperostosis, osteitis (SAPHO) syndrome is a rare disease; however, more and more case reports have been published that increase the awareness of this disorder, especially in children. Clinically it presents as a combination of chronic recurrent multifocal osteomyelitis symptoms and skin manifestations. SAPHO treatment remains a challenge. In most cases, non-steroidal anti-inflammatory drugs are initially used, although a combination with other drugs is preferred. In addition, antibiotics, corticosteroids, bisphosphonates, and disease-modifying anti-rheumatic drugs are usually administered with varied success. There are also promising results from novel biological therapy. This paper emphasizes some non-specific symptoms of the disease, in order to increase the suspicion of SAPHO in all pediatric clinical doctors. We present the case of a 13-year-old boy with severe acne, who was admitted to our hospital due to fever of unknown origin, accompanied by arthralgia of the right ankle and left knee.

## Introduction

Synovitis, acne, pustulosis, hyperostosis, osteitis (SAPHO) syndrome is a rare disorder that is often misdiagnosed. The etiology remains unclear. Genetic, infectious, and immunological factors have been implicated. It can affect any age and is increasingly recognized among pediatric patients [1].

The first described term, chronic recurrent multifocal osteomyelitis (CRMO), refers to a rare chronic musculoskeletal inflammatory disease in children [2]. It is now known that the term SAPHO, first introduced by Chamot in 1987, associates the osteoarticular symptoms with different skin abnormalities [1]. Although in SAPHO the axial skeleton is mostly involved, especially sternoclavicular and sternocostal joints, metadiaphyses of long bones are also affected to a lesser proportion in children [1]. Effective treatment remains a challenge. We present the case of a 13-year-old boy with severe acne, who was admitted to our hospital due to fever of unknown origin, accompanied by arthralgia of the right ankle and left knee.

## Case presentation

**Figure 1:**
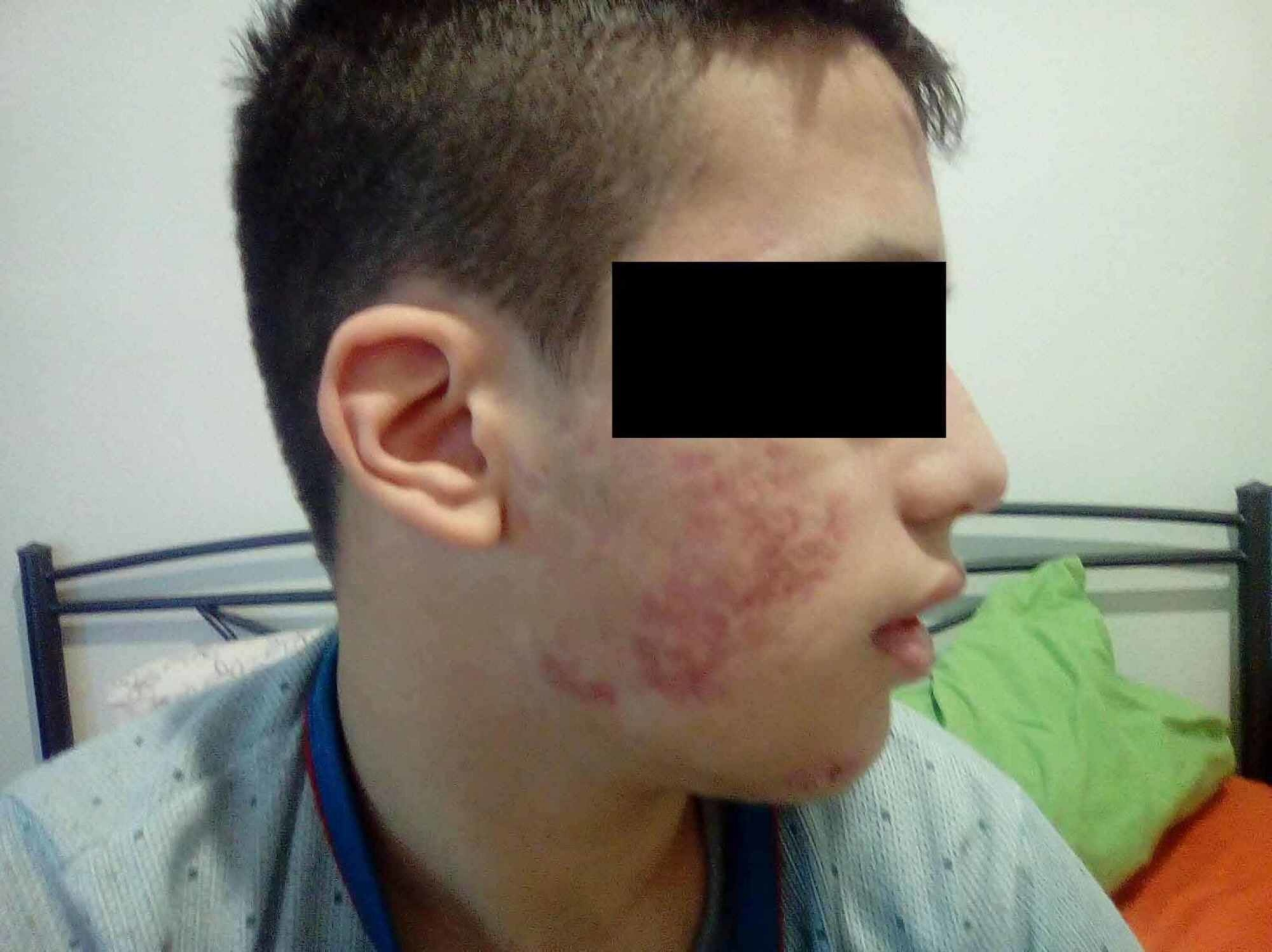
Severe acne of the face

A 13-year-old boy presented to our pediatric emergency department with fever of unknown origin for more than three weeks. The aAccompanying symptoms were pain in the right ankle and left knee, which had started three days before the onset of fever. During physical examination, he experienced severe tenderness upon palpation of his right ankle and only mild tenderness of the left tibial tuberosity. The range of motion was not restricted, and there were no signs of local infection. Other physical findings were severe acne of the face with multiple cysts (Figure 1) and skin lesions on the back. The patient had received treatment with isotretinoin for two months, which had been discontinued due to onset of the fever and joint pain.

**Figure 2:**
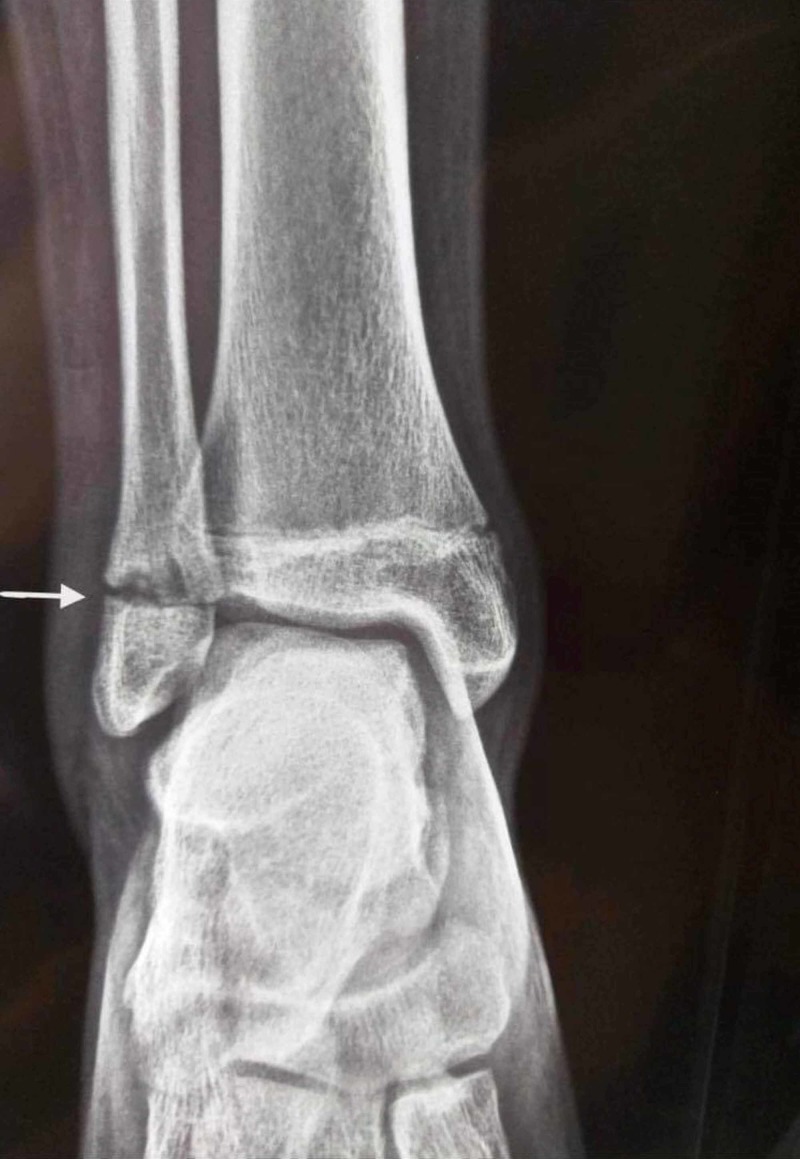
X-ray of the right ankle Osteolytic changes of the right fibula (white arrow)

Blood tests on admission revealed elevated C-reactive protein (82 mg/dL; normal range <5 mg/dL), elevated erythrocyte sedimentation rate (50 mm/hr; normal range 3-13 mm/hr), and mild leukocytosis (14,200 cells/mm^3^). Biochemical test results were normal. Blood and urine cultures showed no bacterial growth. Ultrasonography of the upper and lower abdomen detected a small number of mesenteric lymph nodes. In addition, rheumatoid factor, anti-double-strand DNA, antinuclear antibodies, and antineutrophilic cytoplasmic antibodies were negative. Furthermore, Widal, Rose Bengal, and Mantoux tests were also negative. There was a slight elevation of the C3 complement (2.140 g/L, normal range 0.825-1.800 g/L). In addition, serum amyloid A was markedly elevated (386 mg/L, normal range <6.4 mg/L). HLA B27 antigen was negative. The X-ray of the right ankle revealed an osteolytic region just proximal to the growth plate of the fibula (Figure 2), but there were no pathological findings on the X-ray of the left knee.

**Figure 3:**
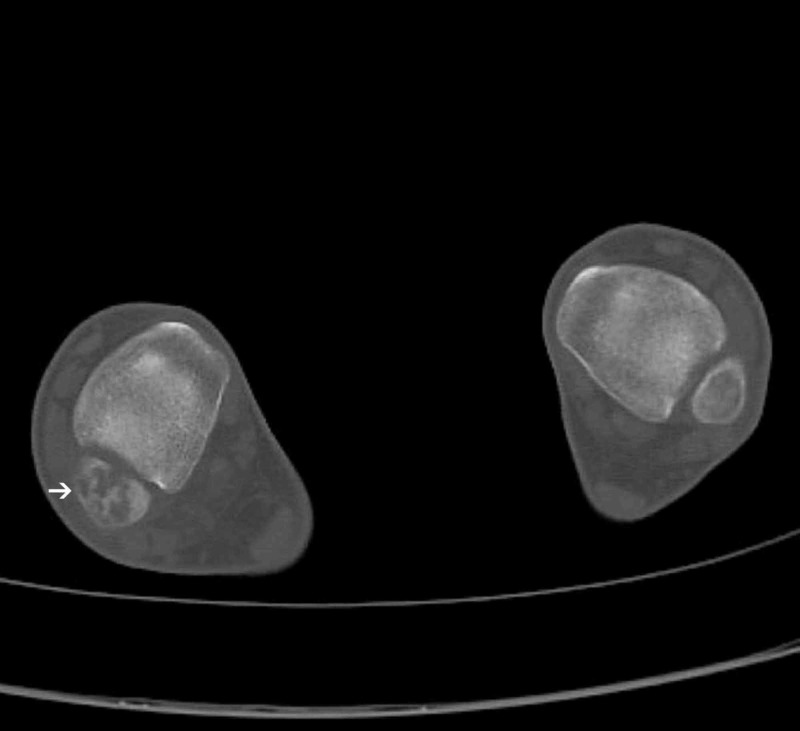
CT scan of the right ankle joint Osteolysis of the right fibula with thinning of the bone cortex on its medial side (white arrow)

CT scan of the ankle revealed osteolytic lesions in the epiphysis and metaphysis of the right fibula with thinning of the bone cortex on its medial side (Figure 3).

**Figure 4:**
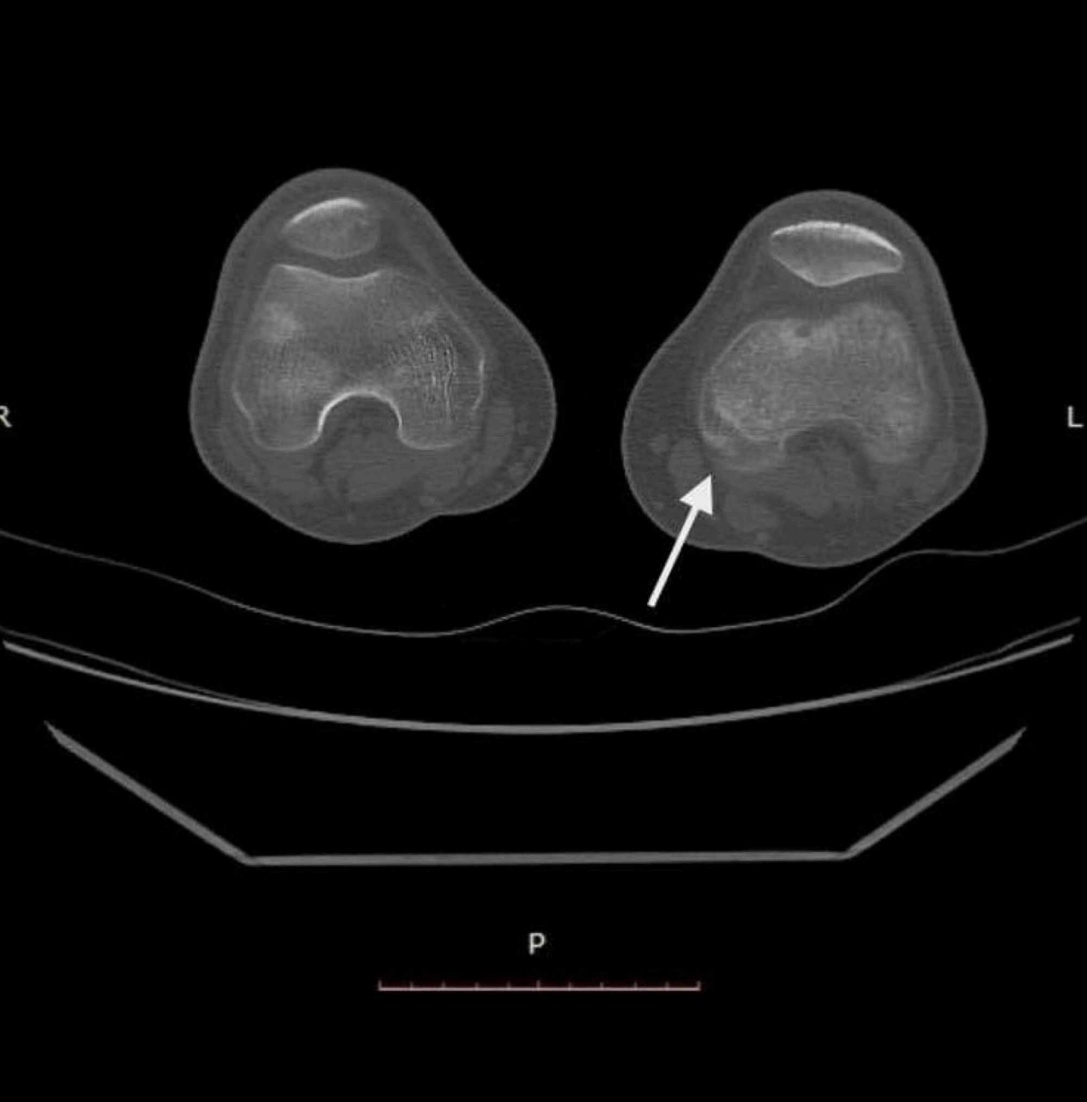
CT scan of the left knee Osteolytic changes in the metaphysis and epiphysis of the left femur (white arrow)

Moreover, CT scan of the left knee revealed osteolytic changes in the metaphysis and epiphysis of the femur (Figure 4) and similarly in metaphysis of the tibia.

**Figure 5:**
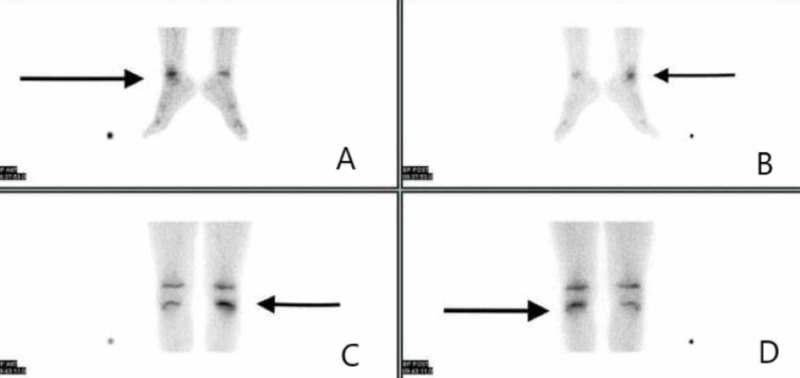
Bone scintigraphy findings (A) Increased uptake on the metaphysis of the anterior side of the right fibula (black arrow). (B) Increased uptake on the metaphysis of the posterior side of the right fibula (black arrow). (C) Increased uptake on the metaphysis of the anterior side of the left tibia (black arrow). (D) Increased uptake on the metaphysis of the posterior side of the left tibia (black arrow)

The Technetium 99m whole-body bone scan revealed increased uptake on proximal metaphysis of the left tibia and distal metaphysis of the right fibula (Figure 5).

Biopsy of the right fibula was performed, and pathology examination revealed findings related to CRMO. Last but not least, a genetic test with whole-exome sequencing analysis was performed; however, the result was negative.

Initially, the patient was treated with intravenous clindamycin, due to suspected osteomyelitis. Non-steroidal anti-inflammatory drugs (NSAIDs) and esomeprazole as gastric protection were initiated after rheumatological consultation. Open biopsy was performed, and the ankle was immobilized with a below-knee cast. The patient was discharged with a subscription of ibuprofen 400 mg three times daily and gastric protection. Suspicion for SAPHO syndrome due to clinical and laboratory findings was high, and one-month post-discharge, adalimumab therapy was initiated.

**Figure 6:**
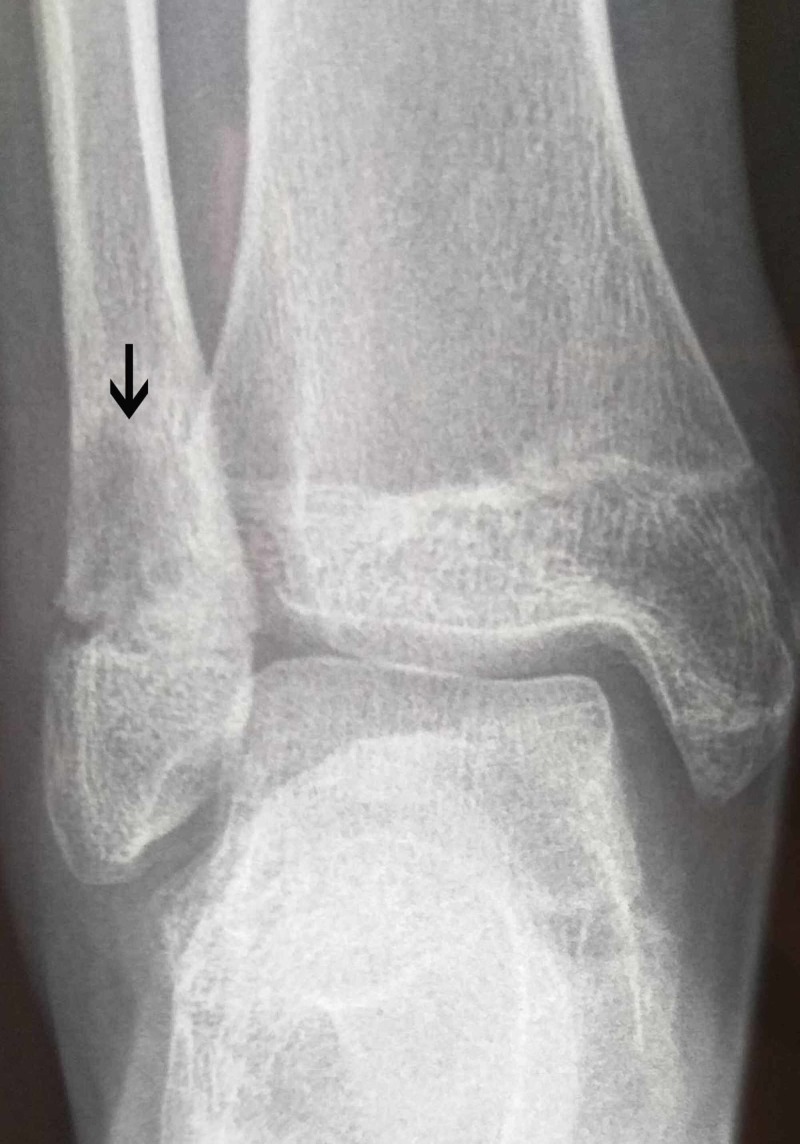
X-ray of the right ankle Three months after the biopsy the radiological image was improved. The osteolytic changes of the right fibula were less evident (black arrow)

The patient was re-examined one-month post-discharge. The cast was removed. On physical examination, there was no tenderness over the right ankle joint and no limitation of movements. Three months after the biopsy, the radiological image of the ankle was improved (Figure 6), and the patient was completely asymptomatic. Acne of the face and back was less severe. In addition, his nine-month follow-up was satisfying.

## Discussion

SAPHO syndrome is rare, especially in children. It is a combination of osteoarticular and dermatological manifestations. The etiology is multifactorial, although researchers have found genes in chromosome 18 (NOD2, LPIN2) that seem to be linked to the disease [1]. In SAPHO patients, the skin is commonly affected by severe acne, palmoplantar pustulosis (PPP), hidradenitis suppurativa, or even with different forms of psoriasis. The appearance of skin symptoms may occur before, after, or simultaneously to bone pain [1]. The report of 10 cases of Beretta-Piccoli et al. noted that there was skin involvement in eight of ten pediatric patients. In three patients, two females with PPP, and one male with psoriasis vulgaris, skin lesions preceded the joint symptoms. In the other five patients, skin disorder appeared in median 0.2 years after the onset of bone lesions [2]. Although skeletal pain is usually the first symptom of SAPHO, Jelušić et al. presented that skin lesions were first manifested in five of six patients and they developed bone symptoms two months to four years later [3]. Divya et al. presented an 18-year-old patient, in whom nodulocystic acne on the face preceded joint pain and systemic symptoms [4].

The treatment of the disease should be focused on the management of bone pain and skin lesions. Unfortunately, there is no targeted therapy since the exact etiopathogenetic mechanisms are not fully understood. Treatment options are empirical and include a variety of medications. First-line agents are the NSAIDs, but their efficacy as monotherapy is limited. Furthermore, corticosteroids, bisphosphonates, and disease-modifying anti-rheumatic drugs (DMARDs) are other options [5-7]. In cases of positive biopsy cultures, antimicrobial therapy is useful. Rozin et al. presented the first reported case of SAPHO syndrome with a positive culture for Staphylococcus aureus, which was treated with sulfamethoxazole/trimethoprim [8]. Also in a study of five patients, treatment with a combination of antibiotics (oral clindamycin) and oral NSAIDs improved the clinical condition in all of the cases. For skin lesions, topical corticosteroids and psoralen ultraviolet A have been administered [9]. Other antibiotics that are mentioned in the literature include doxycycline, azithromycin, and clindamycin [10].

Furthermore, Kerrison et al. reported the use of pamidronate, a second-generation bisphosphonate, in seven girls who were treated for the disease. All patients showed clinical improvement, the use of other medications could be stopped, and there were no significant adverse effects [11]. Also, in a comparative study of children and adults by Skrabl-Baumgartner et al., differences between the two were that children were earlier diagnosed and were affected by severe acne, in comparison to adults, who presented with PPP. Before the exact diagnosis was established, oral/intravenous antibiotics were administered, in combination with cast immobilization, prostaglandin-E infusions, hyperbaric oxygenation, and surgical resection. After diagnosis was established, bisphosphonates were initiated, resulting in nine out of thirteen children experiencing no symptoms [12]. Akçaboy et al. reported a case of an 11-year-old girl, who suffered from SAPHO and, after an unsuccessful combination of NSAIDs-sulfasalazine and prednisolone, was relieved by administration of subcutaneous methotrexate. At a two-year follow-up, she was free of symptoms [13].

For the patients who are unresponsive to bisphosphonates, biologic agents are used [14]. Eleftheriou et al. suggested anti-TNF-α therapy for cases refractory to bisphosphonates in the case presentation of a 15-month-old male with SAPHO syndrome who presented with finger dactylitis. He was initially treated with naproxen, intra-articular corticosteroids, methotrexate, and pamidronate infusions. Sixty-seven months from the initial diagnosis, infliximab therapy was initiated (due to reported feet pain) which was switched to adalimumab subcutaneously. No adverse effects were noted, and the 15-month follow up was good [15]. Also, Wagner et al. described the efficacy of TNF-α blocking agents, infliximab and etanercept, in two patients, who failed to have sufficient clinical improvement with different DMARDs [16]. 

In the case of our patient, antibiotic therapy was initially administered due to suspicion of osteomyelitis. After the biopsy results, treatment with NSAIDs was given, which was also continued at home. One month post-discharge, biologic agent was initiated. His nine-month follow-up is encouraging.

## Conclusions

SAPHO syndrome is rare in the pediatric population which may result in its late diagnosis. It is a challenging disease since symptoms are often non-specific. Furthermore, osteoarticular manifestations and skin lesions may not be present at the same time. The patients may initially be referred to orthopedic surgeons, due to arthralgia and movement limitation. Meticulous clinical examination is mandatory, and extensive laboratory and imaging workup should be performed in the event of a pathological X-ray. Skin manifestations in a patient with any form of osteitis should raise suspicion for SAPHO syndrome, and a rheumatology consultation should be sought. Early recognition of the disease is essential in order to avoid prolonged antibiotic treatment and unnecessary invasive examinations, and to relieve the patient's symptoms. 
